# The Weather and Diseases

**Published:** 1853-09

**Authors:** 


// THE WESTERN JOURNAL.

Third Series.—Vol. XII.—No. III.

LOUISVILLE, SEPTEMBER, 1853.

### THE WEATHER AND DISEASES.

The summer just ended will be long remembered by the citizens of Louisville, as one of the hottest and most healthy that ever passed over them. It would be difficult to find a community in the same latitude numbering so many people where there have been so few cases of sickness as our city has afforded in the last three months. We have spoken heretofore of the temperature of June. July was a temperate month; but during the first half of August the heat was as intense for a few days as it was in June. The thermometer rose to 98° and 99° in the shade. The rains have been seasonable, and on the 7th of the month the city was visited by one of the heaviest showers that we have ever seen fall.

In connection with this unusual salubrity, we ought not to fail to record the fact, that fruit, in all its varieties, has abounded and been consumed by our citizens to an extent never before exceeded. The smaller fruits early in the season, and since then peaches, plums, pears, apples, and melons have been brought to our markets in the greatest profusion. If the old prejudice still lingers in the mind of any one that "fruit years are years of sickness," the experience of the present season must dissipate it. Certainly, no one can ever hope to see Louisville more exempt, in summer, from fevers and affections of the bowels than it has been for months past, and certainly fruit is not likely ever to be more abundant. We feel a peculiar pleasure in stating these facts. There is still a lurking apprehension in the public mind, that fruit is one of the causes of sickness; and a more unfounded prejudice, we are convinced, does not exist. Fruit is eminently conducive to health. Its culture ought to bu encouraged by every friend of his race.

While instances of *coup de soliel* have been so frequent in the city of New-York, scarcely one has occurred here. Probably the following, for the particulars of which we are indebted to our friend, Dr. D. J. O'Reilly, was a case of the kind:

"D. Curran, aged 45 years, employed as a laborer in hoisting cut stone upon a building in this city, was taken suddenly ill at his work, on the afternoon of July 29th, and was conveyed to his residence in a state of extreme prostration. I was immediately called upon to visit him, and found him exhibiting the following symptoms: Face slightly flushed and covered with a cold perspiration, pulse irregular, soft and easily compressible. average beat at the wrist about 95, tongue moist and coated with a light white fur, stomach irritable, with frequent but ineffectual efforts at vomiting, bowels regular, respiration from 25 to 30, cramps in the flexor muscles of the extremities but more especially in one of the legs, mind wandering, occasional muttering delirium, during which the patient seemed to be engaged in his usual avocation, and to converse with his fellow workmen. From all the symptoms I concluded to treat the case as one of nervous irritation, rather than one of active venous congestion of the brain or its membranes, and administered stimulants, and narcotics, combining the latter with a moderate dose of calomel. The medicine seemed to operate in the most favorable manner during the night, and next morning I ordered a mild cathartic, with the effect of producing moderate purgation, after which the patient continued to improve and was able to resume his work in the course of three or four days, but was again attacked, in the same manner, on the evening of the 13th of August, and expired before he could receive any medical aid.

His friends would not permit a post mortem examination."

